# Differences between cultured cortical neurons by trypsin and papain digestion

**DOI:** 10.1002/ibra.12028

**Published:** 2022-03-08

**Authors:** Chang‐Yan Hu, Ruo‐Lan Du, Qiu‐Xia Xiao, Min‐Jian Geng

**Affiliations:** ^1^ Animal Zoology Department Kunming Medical University Kunming Yunnan China; ^2^ Institute of Neurological Disease, West China Hospital Sichuan University Chengdu Sichuan China; ^3^ Department of Anesthesiology Nanchong Central Hospital Nanchong Sichuan China

**Keywords:** cortical neuron, papain, trypsin

## Abstract

The objective of this study was to compare the efficiency of trypsin and papain in neuronal digestion and determine which enzyme is more efficient. Cortical tissues were obtained from Sprague–Dawley (SD) rats. According to the different digestive enzymes, the samples were divided into the trypsin group and the papain group. After being digested by each of the two enzymes, cortical neurons were collected from the samples. Then, the morphology of the cortical neurons was determined. Moreover, the cortical neurons were transfected with the negative control (NC) lentivirus. The transfection efficiency and morphology were determined and compared. Compared with the papain group, cortical neurons in the trypsin group were more in number, had larger cell size, had longer axonal length, and had fewer impurities. The transfection efficiency of the trypsin group (57.77%) was higher than that of the papain group (53.83%). The morphology of neurons that was displayed showed that the cell body of most neurons shrank and became smaller, and the axis mutation became shorter and less in the papain group 6 days after transfection with the NC lentivirus. Trypsin is more efficient in digesting neurons because the neurons digested by this enzyme are more in number, have a larger cell body, longer axons, and greater transfection efficiency.

## Graphical abstract

In the present study, a comparative study of trypsin and papain digestion for primary cortical neurons was conducted. Cortical neurons digested by trypsin were more in number, had larger in cell body size, longer in axonal length, and had fewer impurities. Meanwhile, the transfection efficiency of the lentivirus in the trypsin group was higher than that in the papain group. The morphology of neurons that was displayed showed that the cell body of most neurons shrank and became smaller, and the axis mutation became shorter and less in the papain group 6 days after transfection with the NC lentivirus.

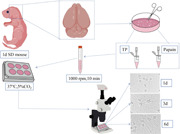

## 1 INTRODUCTION

The brain is a sophisticated command center that has different types of cells. Such complexity hampers studies and investigations into how specific cells affect our health. Therefore, creating an extracorporeal model that shares the exact or similar morphology and function of certain cells is necessary. With advancements in scientific research, in order to better elucidate the mechanism, the research needs to be done not only in living animal (in vivo),  but also using cell culture in−vitro  experiments for verification. The culture of neurons has enabled great convenience in the study of neuron growth, development, and pathophysiology mechanisms in vitro, which is helpful for medical staff and researchers to gain further understanding of a variety of neurological diseases, such as Alzheimer's disease, Parkinson's disease, neonatal hypoxic–ischemic encephalopathy, stroke, cerebral ischemia/reperfusion injury, and so on.^1^, ^2^, ^3^, ^4^, ^5^ Primary culture of neurons can fulfill such demands. Primary cultured neurons are very similar to in vivo neurons, the experimental conditions are stable and controllable, and the experimental environment is relatively simple. In addition, observation and detection can eliminate the interference of many factors such as circulation, body fluids, endocrine, and the blood–brain barrier, which has become an indispensable model tool in neuroscience research. More importantly, the quality of cell culture will directly affect the progress of research work.^6^

Primary culture of neurons refers to the culture of cells or tissues obtained from living organisms in vitro.^7^ When neurons are isolated and cultured from embryonic brain tissue, the neurons will no longer divide and proliferate, which makes neuronal culture very different from other types of somatic cell cultures.^8^ Currently, there are many methods for culturing primary cortical neurons, such as mechanical pipetting, trypsin digestion, and the papain method.^9^, ^10^, ^11^ Cortical neurons can be cultured through enzyme digestion, which enables separation of neurological tissues.^12^ Currently, both trypsin and papain have wide applications in culturing primary neurons.^13^ Both enzymes can be used for digesting neurons, but few studies have compared their efficiency.

In this study, based on the relevant literature, a comparative study of trypsin digestion and papain digestion was conducted with the aim of finding which enzyme is more effective in culturing cortical neurons.

## 2 MATERIALS AND METHODS

### 2.1 Animals

All Sprague–Dawley (SD) rats that were specific pathogen‐free neonates 24  after birth were provided by the Animal Zoology Department of Kunming Medical University.

### 2.2 Pretreatment of culture plates

On the day before the cortical samples were taken, six‐well plates were coated with poly‐l‐lysine (cyagen) at a concentration of 25 μg/ml for 2 h, washed twice with phosphate‐buffered saline, and then dried in an incubator filled with 5% CO_2_ at 37°C for later use.^9^, ^14^

### 2.3 Primary culture of cortical neurons

Newborn SD rats were killed by decapitation. The cortical tissue was isolated and placed in prerecord Dulbecco's modified Eagle's medium (DMEM) high‐glucose medium (BI). The tissue was minced to 1 mm^3^ using micro scissors and then transferred to a 2 ml Eppendorf tube. In the trypsin group, 500 μl of 0.25% trypsin (containing EDTA; Gibco) was added to the tube, and the tissue was digested in an incubator filled with CO_2_ at 37°C for 10 min, which was shaken every 5 min. In the papain group, 1 ml of DMEM high‐glucose medium and 50 μl of papain (diluted 20 times) were added to the tube for digestion. Afterward, 500 μl of inoculation medium (DMEM high‐glucose + 10% serum + 1% penicillin–streptomycin solution) was added to stop digestion. The tissue was gently pipetted 30 times with a Pasteur tube, and let it stand for 2 min. Next, the supernatant suspension was filtered through a 70 μm cell strainer to a 15 ml tube and centrifuged for 10 min at 1000 rpm/min. One milliliter of inoculation medium was added after removing the supernatant, and the cell suspension was resuspended by pipetting 20 times. After the volume was adjusted to 3 ml, the cells were counted, inoculated in a six‐well plate with 1 × 10^6^/well, and cultured in an incubator filled with 5% CO_2_ at 37°C. After culturing for 4 h, the medium was replaced with neuron medium (Neurobasal + 2% B27 + 1% glutamine + 0.5% penicillin–streptomycin solution).^15^, ^16^, ^17^ Photographs were taken using an inverted phase‐contrast microscope. Five pictures were taken in each well on different days to count the number of cells. Five cells were taken from each picture to measure cell body size and axonal length. Neurons were cultured for 6 days, and five images were selected from each well to count the number of impurities. GraphPad 8.0 was used to construct graphs to compare the effects of trypsin and papain digestion on the number of cortical neurons and impurities, cell body size, and axonal length.

### 2.4 Lentiviral transfection and transfection efficiency calculation

On the third day of neuron inoculation, the medium was replaced with the neuron medium without glutamine and penicillin–streptomycin solution. The required volume of the negative control (NC) lentivirus was calculated with the multiplicity of infection (MOI) = 5, lentivirus volume = MOI × cell number/lentivirus titer. After that, transfection reagent A (diluted 50 times) was added to each well of neurons. The lentivirus was added to the neurons. Ten hours after transfection, the medium was replaced with the neuronal medium without glutamine and penicillin–streptomycin solution. Three days after lentivirus transfection, the transfection efficiency was recorded under a fluorescence microscope. Transfection efficiency = green fluorescent cells/total number of cells. The morphology of neurons was observed 6 days after transfection.^18^, ^19^, ^20^

### 2.5 Statistical analysis

All data in the experimental process are presented as mean ± standard deviation (SD). SPSS 21.0 was used for statistical analysis. The difference between the trypsin and papain groups was analyzed using a *t *test of independent samples. *p* < 0.05 indicated a significant difference.

## 3 RESULTS

### 3.1 Primary cortical neural culture process

**Figure 1:**
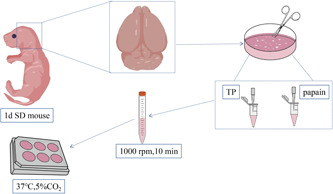
Illustration of culture and observation of cortical neurons. d, day; min, minutes; SD, Sprague–Dawley; TP, trypsin [Color figure can be viewed at wileyonlinelibrary.com]

After disinfection with 75% ethanol, the SD rats (1 day old) were killed by decapitation. The cortical tissue was taken, digested, and inoculated with 0.25% trypsin and papain. The growth of cortical neurons was observed on Days 1, 3, and 6 as shown in Figure 1.

#### 3.1.1 Morphological changes of primary cortical neurons on Days 1, 3, and 6

**Figure 2:**
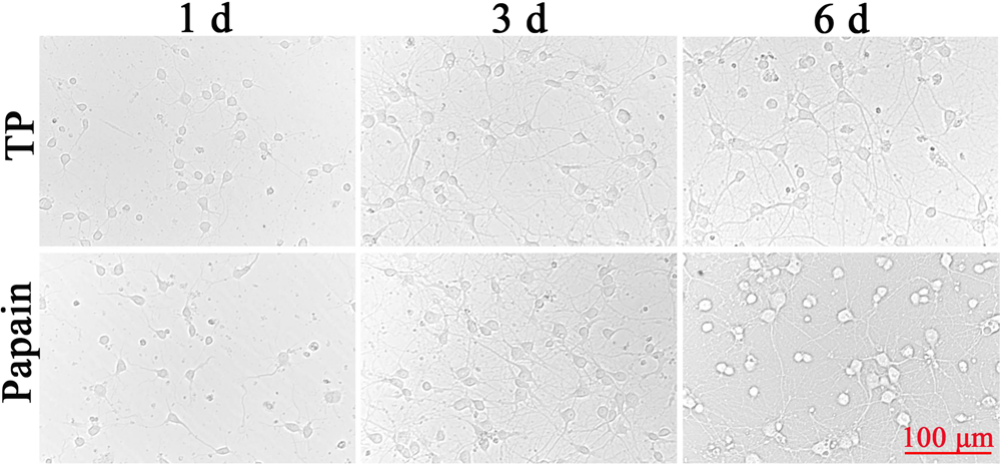
Morphology of primary cortical neurons on Days 1, 3, and 6. Scale bar = 100 μm. d, days; TP, trypsin [Color figure can be viewed at wileyonlinelibrary.com]

The cortical neurons were round, translucent, and refractive when they were just seeded. After seeding for 4 h, most of the neurons adhered to the wall. The cells were round, small, and transparent, and had not yet grown synapses. One day after inoculation, a small number of synapses grew out and the synapses were short and few. Three days after inoculation, the synapses became longer and increased in number, and the cell bodies were enlarged. Six days after inoculation, the synapses became longer and increased in number, forming a network, and the cell body also increased (Figure 2).

#### 3.1.2 Neuron numbers, cell body size, axonal length, and number of impurities in cortical neurons digested by trypsin and papain

**Figure 3:**
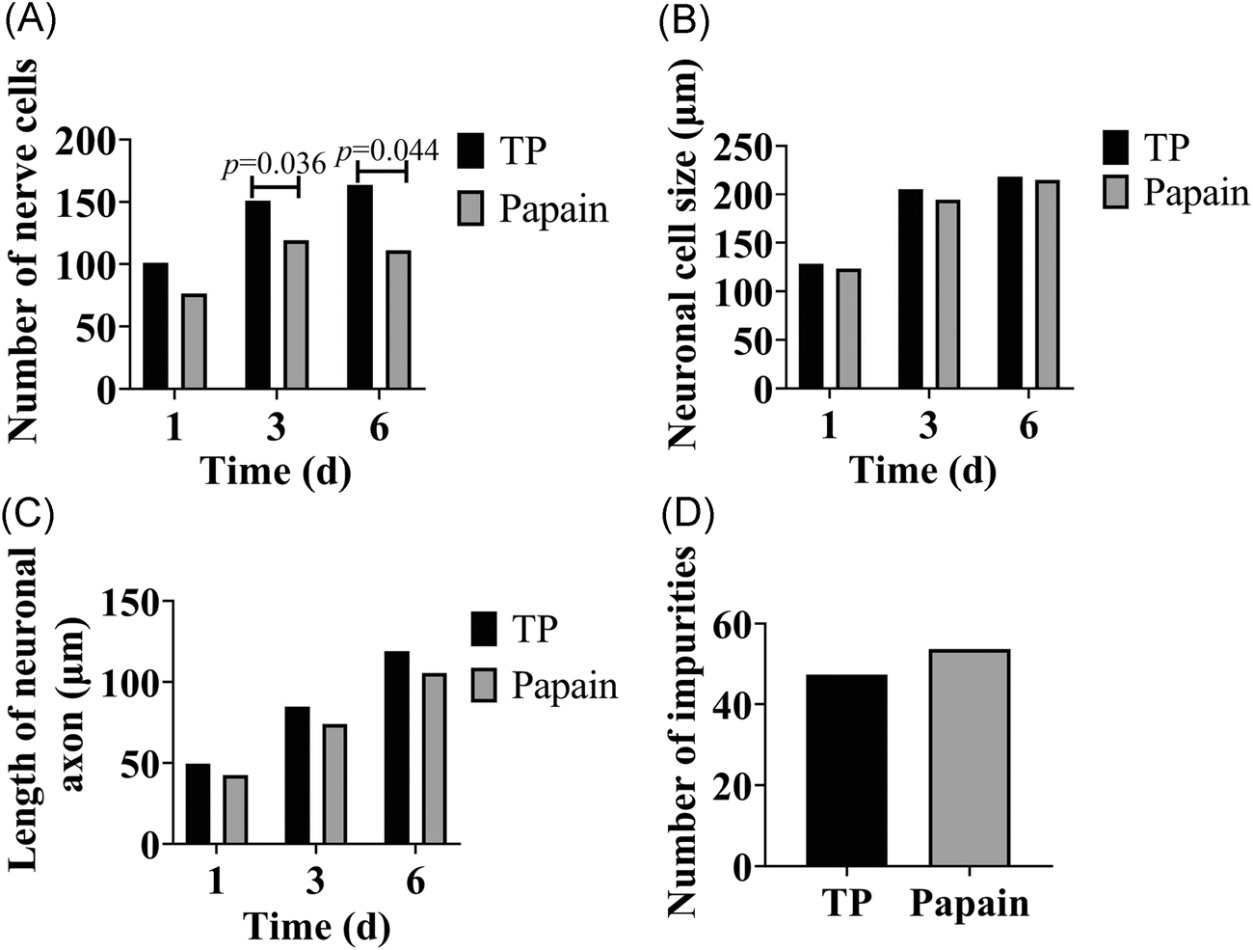
Neuron numbers, cell body size, and axonal length on Days 1, 3, and 6 and number of impurities on Day 6. (A) Neuron number; (B) cell body size; (C) axonal length; and (D) number of impurities. d, days; TP, trypsin

The number of cells, cell body size, and axonal length were compared after inoculation with trypsin and papain on Days 1, 3, and 6, respectively. The results showed that the difference in the number of cells digested by trypsin and papain was not statistically significant on Day 1 (*p* > 0.05), whereas the number of cells digested by trypsin was higher than that in the papain group on Day 3 (*p* = 0.036) and Day 6 (*p* = 0.044) (Figure 3A). In addition, the neuronal cell size, the length of neuronal axons, and the number of impurities were not different between the trypsin and papain groups (*p* > 0.05) (Figure 3B–D). Nevertheless, as can be seen from Figure 3, the number of cortical neurons digested by trypsin was higher than that digested by papain, the cell body was larger, and the axon length was longer. It could be seen that cortical neurons were digested by papain for 6 days, and there were more impurity spots digested by papain than that digested by trypsin.

### 3.2 Morphology of primary cortical neurons on the third day after transfection with the NC lentivirus

**Figure 4:**
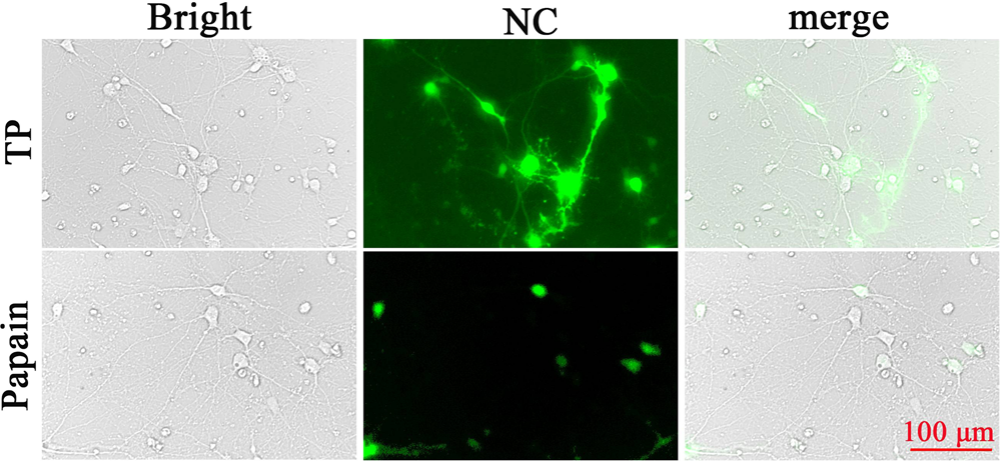
Primary cortical neurons transfected with the NC lentivirus on Day 3. Scale bar = 100 μm. NC, negative control lentivirus; TP, trypsin [Color figure can be viewed at wileyonlinelibrary.com]

On the third day after inoculation, the cortical neurons were transfected with the NC lentivirus. The figures were obtained using a fluorescence inverted microscope on the third day after transfection. The result showed that the cortical neurons transfected with the NC lentivirus that were digested by trypsin were higher than those digested by papain, and the green fluorescence was brighter in the trypsin group (Figure 4).

#### 3.2.1 Transfection efficiency of primary cortical neurons transfected with the NC lentivirus on Day 3

**Figure 5:**
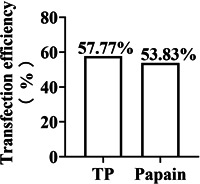
Transfection efficiency after primary cortical neurons transfected with the NC lentivirus on Day 3. TP, trypsin

On the third day after inoculation, cortical neurons were transfected with the NC lentivirus. The transfection efficiency of neurons digested by trypsin was 57.77%, and the transfection efficiency of neurons digested by papain was 53.83%. Although the transfection efficiency between the trypsin and papain groups was not statistically different (*p* > 0.05), the transfection efficiency of the trypsin group was higher than that of the papain group (Figure 5).

### 3.3 Morphology of primary cortical neurons on Day 6 after transfection with the NC lentivirus

**Figure 6:**
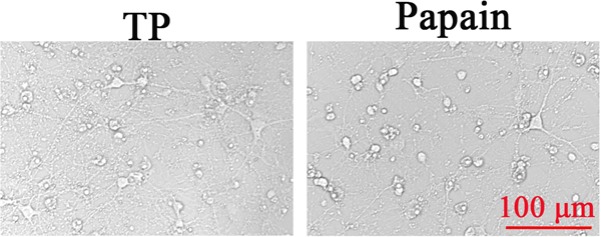
Primary cortical neurons transfected with the NC lentivirus on Day 6. Scale bar = 100 μm. TP, trypsin [Color figure can be viewed at wileyonlinelibrary.com]

Cortical neurons were transfected with the NC lentivirus for 6 days; that is, the 12th day after cell inoculation, the morphology of neurons was collected. It was found that the cell body of most neurons shrank and decreased in number, and the axis mutation became shorter and decreased in number in the papain group compared with those digested with TP (Figure 6).

## 4 DISCUSSION

In this experiment, trypsin and papain were used to digest and culture primary cortical neurons. It was found that the number of cortical neurons was higher, the cell body was larger, the axonal length was longer, and the number of impurities was lower in the trypsin group compared with the papain group. The lentiviral transfection efficiency in the trypsin group was higher than that in the papain group. Six days after lentivirus transfection, observation of the morphology of neurons showed that the cell body of most neurons shrank and decreased in number, and the axis mutation became shorter and decreased in number in the papain group compared with those digested with TP.

As neurons find more applications in multi‐field and multi‐level research including cell function, neurodevelopment, degenerative diseases, and neuropharmacological toxicology, culture of primary cortical neurons is becoming increasingly significant.^21^, ^22^, ^23^, ^24^, ^25^ Primary neuron culture is an important basic model for the study of neurological diseases, and has become an important technical means to study neuron development, differentiation, and the mechanism of neurological diseases.^26^, ^27^, ^28^ Cortical culture neurons enable understanding of the mechanism, pathology, and therapeutics in degenerative conditions including Alzheimer's disease, Parkinson's disease, Huntington's disease, and so on.^29^

This experiment compared two methods of enzymatic digestion for primary neuronal cultures. Based on the experimental results, the primary cortical neurons cultured by trypsin digestion were superior to the cortical neurons cultured by papain digestion in terms of morphology and lentiviral transfection efficiency. In the primary culture of neurons, the selection of appropriate enzymes, the number and concentration of enzymes, and the digestion time of enzymes are the key steps in the digestion of tissue blocks into single cells.^30^ Trypsin digestion is a biochemical and chemical separation technique. The main function of trypsin is to digest the intercellular substance, so that the cells disperse. If the digestion time is too short, the cells are not easy to disperse, the cell clumps are large, and the cells do not easily adhere to the wall, which is obviously unfavorable for the cells that grow adherently. If the digestion time is too long, the physical and chemical properties of the cell membrane surface will deteriorate, and the cells will not easily adhere to the wall or grow poorly.^31^ Trypsin digestion is related to the pH, concentration, temperature, and digestion time. The concentration and the duration of digestion will directly affect the production and activity of cells. The literature shows that when 0.25% trypsin is used for 5–10 min of digestion, neurons grow most vigorously.^32^, ^33^ Cell processes are abundant. Monolayer cells are evenly dispersed on the culture plate, showing the growth trend for the reticulum form.

To sum up, from the experimental results, we could conclude that trypsin is more efficient in the primary culture of cortical neurons. Hopefully, this study will pave the way for further advanced research on trypsin in neuronal digestion.

## CONFLICTS OF INTEREST

The authors declare no conflicts of interest.

## ETHICS STATEMENT

All experiments in the present study were approved by the Animal Experiment Ethics Committee of Zunyi Medical University, and the approval number is [2020] 2‐097.

## AUTHOR CONTRIBUTIONS

Qiu‐Xia Xiao came up with the idea of the study. Chang‐Yan Hu and Ruo‐Lan Du completed the experiment and analyzed the data. Min‐Jian Geng performed the document retrieval, and revised and polished the manuscript.

## Data Availability

Data of our study are available on reasonable request.
